# Maternal Alcohol Consumption during Pregnancy and Early Age Leukemia Risk in Brazil

**DOI:** 10.1155/2015/732495

**Published:** 2015-05-18

**Authors:** Jeniffer Dantas Ferreira, Arnaldo Cézar Couto, Mariana Emerenciano, Maria S. Pombo-de-Oliveira, Sergio Koifman

**Affiliations:** ^1^Environment and Public Health Postgraduation Program, National School of Public Health, Oswaldo Cruz Foundation (FIOCRUZ), Rua Leopoldo Bulhões 1480, 21041-210 Rio de Janeiro, RJ, Brazil; ^2^State University Center of the West Zone (UEZO), Rua Manuel Caldeira de Alvarenga 1203, 23070-200 Rio de Janeiro, RJ, Brazil; ^3^Pediatric Hematology-Oncology Program, Research Center, Instituto Nacional de Câncer (INCA), Rua André Cavalcanti 37, 20231-050 Rio de Janeiro, RJ, Brazil

## Abstract

*Objectives*. To investigate the association between the maternal alcohol consumption during pregnancy and early age leukemia (EAL) in offspring.* Methods*. Datasets were analyzed from a case-control study carried out in Brazil during 1999–2007. Data were obtained by maternal interviews using a standardized questionnaire. The present study included 675 children (193 acute lymphoid leukemia (ALL), 59 acute myeloid leukemia (AML), and 423 controls). Unconditional logistic regression was performed, and adjusted odds ratios (adj. OR) on the association between alcohol consumption and EAL were ascertained.* Results*. Alcohol consumption was reported by 43% of ALL and 39% of AML case mothers and 35.5% of controls'. Beer consumption before and during pregnancy was associated with ALL in crude analysis (OR = 1.54, 95% CI, 1.08–2.19), although in adjusted analysis no statistical significance was found. For weekly intake of ≤1 glass (adj. OR = 1.30, 95% CI, 0.71–2.36) and ≥1 glass/week (adj. OR = 1.47, 95% CI, 0.88–2.46) a potential dose-response was observed (*P* trend < 0.03).* Conclusion*. This study failed to support the hypothesis of an increased risk of EAL associated with maternal alcohol intake during pregnancy, neither with the interaction with tobacco nor with alcohol consumption.

## 1. Introduction

Acute leukemia in early childhood is seldom, mainly those cases diagnosed within the first year of life—infant leukemia (IL) [1, 2]. IL deserves special attention because this group is biologically and clinically distinct from leukemia in older children [3]. The somatic gene mutations in clonal cells, for instance,* MLL* gene rearrangements (*MLL-r*), constitute the biological basis of this hematopoietic malignancy that arises during fetal life [4, 5].

Although the etiology of the majority hematopoietic malignancies in children remains largely unknown, Down syndrome [6, 7] and exposure to ionizing radiation [8] and certain chemotherapeutic agents [9, 10] are associated with increased risk of childhood acute leukemia. Previous researches demonstrated that for some childhood leukemia types the causality factors are likely to be multiple and associated with leukemia subtype-specific, combining environmental exposures and genetic susceptibility modulation risk [11, 12].

Several studies conducted in the last decade demonstrated a positive association between childhood leukemia and maternal alcohol consumption during pregnancy [13–17] leading to the premises that maternal alcohol drinking during pregnancy could cause DNA damage during the preconceptions in gametes cells or during pregnancy in fetal cells. Ethanol was established as a teratogen substance that produces pre- and postnatal growth deficiency according to experimental models [18]. Recently, additional epidemiological observations confirmed the previous studies, but findings with low risk association estimates could be a consequence of methodological approaches [19–24].

Several chemical and biological mechanisms likely contribute to the damaging effects of alcohol exposure on the developing fetus. The toxic metabolite acetaldehyde resulting by the break-down of alcohol in the liver and other tissues plays a major role in the tumor-promoting effect demonstrated by animal models [25, 26]. Transplacental crossover alcohol metabolites were measured and similar fetal and mother alcohol concentration rates were observed, leading to the conclusive evidence that the amniotic fluid acts as a reservoir of alcohol toxic metabolites [27, 28]. Other suggested mechanisms for carcinogenic pathways include cell death by apoptosis, increased oxidative stress, and facilitation of cellular entry for other carcinogens [29].

In order to test the hypothesis that maternal exposure to alcohol consumption would be associated with early age leukemia (EAL), we analyzed acute lymphoblastic (ALL) and myeloid (AML) cases included in the Brazilian Collaborative Study Group of Infant Leukemia (BCSGIAL). The joint effects of maternal smoking exposure and alcohol consumption as addictive effects were also tested.

## 2. Materials and Methods

### 2.1. Study Population

Cases and controls were assessed throughout a multicenter study “Multi-Institutional Study of Infant Leukemia: Contribution of Immunomolecular Markers in Distinguishing Different Etiopathogenic Factors” that focuses on the investigation of EAL. It was a hospital-based study in which the participants were ascertained from different Brazilian regions [31, 32].

### 2.2. Case and Controls Ascertainment

Eligible cases were children with acute leukemia (ALL or AML) aged ≤24 months at the diagnosis, confirmed by cell morphology, immunophenotyping profile, and standard cytogenetic-molecular methods [33]. The controls were selected children with nonmalignant diseases that were attended in hospitals where cases were recruited and also from pediatric care centers in the same cities. They were frequency matched with leukemia cases according to age (≤24 months) and enrolled from the same geographic areas where cases were diagnosed. The reasons for clinical assistances were viral infections and parasitic diseases (*n* = 124, 29.4%); nonmalignant hematological diseases (*n* = 83, 19.6%); asthma and bronchitis (*n* = 43, 10.2%); hemangioma (*n* = 40, 9.4%); severe diarrhea (*n* = 39, 9.2%); cardiovascular diseases (*n* = 25, 5.8%); and other nonmalignant conditions (*n* = 69, 16.4%).

The variables elected for the present analysis were obtained in a dataset built from 1999 to 2007. As soon as the diagnosis of acute leukemia was established, the maternal exposure information was obtained through questionnaires. After the written informed consent was signed, a face-to-face interview was applied to mother of cases and controls. Previous data about different maternal exposures and smoking during pregnancy used herein as a basis for interaction analysis are described elsewhere [30, 32]. The content of questionnaires included data about family income, maternal age at child birth, education level, illness previous history to conception, medication use, occupation, personal habits, and the child's birth characteristics. The exposure assessment to smoking exposure was determined by the qualitative analysis (yes/no) during the three months before the index pregnancy and the three trimesters of the pregnancy, as well as after birth during the breastfeeding period. Levels of pregnancy smoking were categorized as nonsmokers; moderate smokers (1–20 cigarettes/day); heavy smokers (≥20 cigarettes/day) [30].

Like the exposure assessment to maternal alcohol consumption, mothers were asked whether they had ever drunk alcohol (yes/no), occasionally, on a regular basis, during the three months before the index pregnancy, and during pregnancy. Then, further questions were also collected to elucidate the frequency and amount of beverage consumption by number of wine glasses, beer, or spirit drinks per week. Answers were classified as abstainers (0 glasses/week), occasional drinkers (by alcohol consumption less than one glass/week), and frequent drinkers (alcoholic intake more than one glass/week).

### 2.3. Exclusion Criteria

Children with genetic syndromes, myelodysplasia, malignant tumor, adoptive parents, or unknown biological mothers were not eligible to the study (cases and/or controls groups). The frequency rate of acceptance of invited mothers (cases and controls) was 96% and 95%, respectively [32].

### 2.4. Ethical Aspect

This study used primary data obtained from the project “Multi-Institutional Study of Infant Leukemia: Contribution of Immunomolecular Markers in Distinguishing Different Etiopathogenic Factors.” This investigation was approved by the Research Ethics Committee of the Instituto Nacional de Câncer (CEP #005/06).

### 2.5. Statistical Analysis

The sample size calculation was performed considering the percentage of exposed controls of the same exposure to alcohol casual women in childbearing age (36%, as in the Brazilian National Antidrug Secretary Survey), the ratio of 2 controls per case, 80% power of study, with a confidence interval 95%. Unconditional logistic regression was performed to estimate the magnitude of association between maternal alcohol consumption and EAL, being the respective odds ratios (OR) and their 95% confidence intervals (CI) ascertained after adjustment for birth weight (<4,000 g, ≥4,000 g), child's ethnicity (whites or non-whites), maternal age at index birth (<35 years, ≥35 years), maternal education level (≤8 years, >8 years), and oral contraceptive intake during pregnancy (no use, use during pregnancy), previously identified as confounders in the studied dataset [32]. To test the interaction between maternal alcohol consumption and tobacco smoking and the risk of EAL, statistical assessment of effect modification was performed on a multiplicative model by fitting models containing both main effects (smoking and alcohol consumption) and their cross-product terms nested models adjusted for the confounders mentioned above. Assuming independence for both maternal smoking and drinking, the periods during pregnancy were considered as independent variables in the model, referring to a baseline category of abstainers' drinkers and nonsmokers [17].

## 3. Results

The demography distribution of cases and controls is shown in Table 1. There were 116 IL cases (46.0%), a higher proportion of whites observed among all EAL cases (67.5%) than controls (36.2%), *P* < 0.01. The majority of EAL cases (61.95%) and controls (56.0%) were enrolled in the Southeastern cities, with the Northeast cities running second, respectively, 20.6% and 24.1%. Mothers of cases were older than mothers of controls (*P* < 0.01). Maternal levels of education were higher among cases (*P* < 0.01).

Maternal alcohol consumption before (3 months of preconception) and during pregnancy was evaluated as potential risk factor for EAL as shown in Table 2; 150 out of 423 mothers of controls (35.5%) had reported alcohol consumption, either preconception or during pregnancy, whereas 106 out of 252 EAL mothers (42.1%) reported use of alcoholic beverages without differences between mothers of ALL and mother of AML. Maternal alcohol intake both before and during pregnancy was observed in 55% of ALL and 48% of AML cases (kappa *P* value < 0.001 for both). Maternal beer consumption less than 1 glass/week during preconception significantly increased the risk for ALL as crude OR = 1.84, 95% CI (1.12–3.04). All adjusted OR analysis performed to test alcohol exposure during pregnancy and EAL demonstrated no statistically significant results. However, an adj. OR = 1.30 (95% CI 0.71–2.36) for weekly beer intake of ≤1 glass and adj. OR = 1.47 (95% CI 0.88–2.46) for >1 glass show a *P* trend < 0.03 in the ALL subtype. Mothers of ALL cases have not reported spirits consumption during pregnancy. Depending on the type of reported alcoholic beverage consumption in the preconception, risk estimates were slightly more pronounced for spirits in AML, adj. OR = 3.61 (95% CI 0.83–15.7), than for beer, adj. OR = 1.13 (95% CI 0.60–2.14), and other beverages, adj. OR = 2.16 (95% CI 0.74–6.35). According to maternal alcoholic consumption in the same period and ALL development in the offspring, an adj. OR = 1.36 (95% CI 0.91–2.03) was observed for reported beer consumption, adj. OR = 0.84 (95% CI 0.22–3.29) for spirits consumption, and adj. OR = 1.48 (95% CI 0.74–2.96) for other beverages consumption.

For mothers of children with ALL (age stratum ≤ 11 months) who reported consumption of any alcoholic beverages before or during pregnancy an increased OR was observed (adj. OR = 1.29, 95% CI 0.73–2.27), although not statistically significant (Table 3). For alcoholic consumption in the preconception period, the risk estimate was an adj. OR = 1.56, 95% CI 0.88–2.79, and an adj. OR = 1.49, 95% CI 0.77–2.89, during pregnancy. In the stratum of children aged 12-23 months, the highest estimate in cases of AML, was observed for the maternal intake of any alcoholic beverages before or during pregnancy (adj OR = 1.49, 95% CI 0.61 to 3.61).

To evaluate the interaction between maternal alcohol consumption and tobacco smoking and the risk of EAL, a logistic regression analysis was performed and results are shown in Table 4. The OR magnitude of exposure did not vary between models, which resulted in nonstatistically significant results to the overall models analyzed.

## 4. Discussion

Several studies demonstrated a positive association between maternal alcohol consumption during prenatal or pregnancy and childhood leukemia [15–17, 21, 22, 34]. Alcohol consumption during pregnancy may affect fetal cells and is consequently associated with several health hazards, including miscarriages, fetal distress, prematurity, malformations, fetal growth retardation, infections, and neurological and respiratory sequels, in addition to intellectual disabilities [35, 36]. This study was not able to determine the strength or direction of any association with maternal alcohol intake. Beer was the beverage most commonly consumed by mothers in this study. Other than beer, few mothers reported high intake levels of spirits and wines. A slight increase in ALL risk following the amount of beer consumption at preconception period was observed, adj. OR = 1.30 (95% CI 0.71–2.36) for weekly intakes of ≤1 glass and adj. OR = 1.47 (95% CI 0.88–2.46) for >1 cup, *P* trend < 0.03. Data from Brazilian Antidrug Secretary Survey indicated that 9–12% of women between 18 and 44 years of age reported alcohol consumption on a regular basis and 38–44% of women in the reproductive age were abstainers. According to this survey, beer is the most popular beverage choice, pointed by 58% of Brazilian women, followed by wine (34%) and spirits (14%) [37]. Of note, the overall frequencies of abstainers mothers in this epidemiological study were 59.8%, and similar to those reported in population-based surveys [37], beer was by far the preference. The different patterns and types of alcohol drinks consumed according to distinct socioeconomic status in Brazil should be considered; maternal wine consumption is relatively low compared to other studies that provided this information [22, 23].

This null association between maternal alcohol consumption and EAL risk regardless of all the period of exposure during pregnancy is consistent with the literature data, similar to overall childhood risks, which pointed out an inverse association especially according to wine intake, OR = 0.7 (95% CI 0.5–0.9) [22, 38, 39]. Slater et al. reported statistically significant inversed association between maternal alcohol use during pregnancy and IL [34]. In the Canadian, French, and Australian studies, wines are the beverage most consumed. Milne et al. also observed *U*-shaped associations with paternal alcohol consumption in the year before pregnancy, by reduced risk at moderate levels of wine and beer consumption and increased risk associated with high levels of beer intake [23]. Wines contain antioxidant compounds, such as polyphenols, that possibly accomplish the protective effect of DNA damaging of ethanol and acetaldehyde [23]. Nonetheless this speculation is in opposite direction to the causative effect of polyphenols in IL proposed [3]. IL is strongly associated with* MLL-r*, mainly in ALL cases diagnosed before the first year of life [31]. The confirmation of the same* MLL-r* by retrospective analyses of neonatal blood has led to the proposal that transplacental exposure to topoisomerase-II inhibitors during pregnancy would be one of the causation factors of IL [3, 32, 40]. Based on experiments that demonstrated block of topoisomerase-II function by some substances, including phenols, which inhibit the resealing of broken DNA-strand ends, the formation of* MLL* translocations would be associated with exposures to such substances [41, 42].

Alcohol drinking is a behavior that often accompanies tobacco smoking, and its role in childhood cancer causality is scarce [16, 20]. The interaction between of maternal alcohol consumption and tobacco smoking as addictive effect in the risk of EAL was tested. The risk magnitudes for a model assuming effect modification were compared with the baseline model assuming independence of effects, but no effect modification was observed in strength of the associations.

This analysis has some limitations as consequence of case-control study in such rare settings. The hospital-based case-control study design may introduce selection bias depending on the chosen comparison groups [43]. Therefore, we recruited controls with a variety of indications for hospitalization and enrolled controls from general hospitals in the same cities, though not necessarily the same hospitals, in which the cases were diagnosed. As in the majority of the case-control approach, the analysed data of variables were dependent on level of perception of maternal report (more accurate in mothers' cases) for variables such as drug and/or tobacco use (that might cause their child leukemia) causing recall bias. Some possible explanation for the imprecise exposure estimates could be that some exposures were possibly being underreported. Sample size was limited mainly to infant AML and for stratified analysis based on* MLL* status. The reduced numbers of infants with low frequency of maternal exposures report make OR unstable, thus resulting in imprecise estimates of association. Another weakness of our study are the data on paternal smoking before and during pregnancy were not collected during the interviews.

On the other hand, the study has strengths regarding the large series of IL cases, given they are rare, compared with other studies that have tested childhood leukemia in older children.

Thereby, data from the use of tobacco smoking in this study did not show evidence of a modification effect by concomitant maternal use of alcohol and tobacco during pregnancy. In this regard, recently, our group demonstrated the increased risk association between maternal smoking and EAL with* MLL-r* modulated by genetic susceptibility [44]. The significant associations found could guide the design of other observational studies in childhood leukaemia, emphasizing the genetic susceptibility in the mechanistic pathway leading to leukaemia in early childhood.

## 5. Conclusion

This study does not support the hypothesis of an increased risk of EAL associated with maternal alcohol consumption during pregnancy. Additionally, no effect modification was observed in strength of the associations with maternal tobacco and smoking. Nevertheless, parents should be advised to limit alcohol intake when planning a pregnancy due to the premise that alcohol metabolites cause DNA damage in gametes and fetal cells according to experimental models.

## Figures and Tables

**Table 1:** Distribution of selected maternal and child demography of early age leukemia and controls, Brazil, 1999–2007^*^.

	Cases^**^ *N* (%)	Controls *N* (%)	*P* value
Age			
≤11 mo.	116 (46.0)	255 (60.2)	<0.01
12–24 mo.	136 (54.0)	168 (39.8)	
Gender			
Males	130 (51.6)	226 (53.4)	0.643
Females	122 (48.4)	197 (46.6)	
Birth weight			
<4,000 g	234 (92.8)	393 (93.0)	0.470
>4,000 g	16 (6.2)	21 (5.0)	
Missing	2 (0.8)	9 (2.0)	
Ethnicity			
Whites	170 (67.5)	153 (36.2)	<0.01
Non-whites	77 (30.5)	256 (60.5)	
Missing	5 (2.0)	14 (3.3)	
Place of birth			
Northeast	52 (20.7)	101 (24.0)	0.552
Midwest	18 (7.1)	31 (7.3)	
Southeast	155 (61.5)	238 (56.2)	
South	27 (10.7)	53 (12.5)	
Maternal age^a^			
<18 years	8 (3.2)	60 (14.2)	<0.01
18–24 years	91 (36.1)	182 (43.0)	
25–34 years	117 (46.4)	145 (34.2)	
>35 years	36 (14.3)	36 (8.5)	
Maternal education			
<8 years	81 (32.1)	206 (48.6)	<0.01
>8 years	146 (57.9)	209 (49.4)	

Total	252 (100)	423 (100)	—

^a^Maternal age at child delivery; *N*, number of cases; mo. = months.

^*^Data presented in Ferreira et al., 2012 [30]; ^**^including acute lymphoblastic leukemia, *n* = 193, and acute myeloid leukemia, *n* = 59.

**Table 2:** Maternal alcohol consumption preconception and/or during pregnancy, early age leukemia, and control mothers, Brazil, 1999–2007.

Maternal alcohol drinking	Controls *n* (%)	ALL *n* (%)	AML *n* (%)	ALL		AML	
				OR (95% CI)	Adj. OR^a^ (95% CI)	OR (95% CI)	Adj. OR^a^ (95% CI)
No	261 (61.7)	107 (55.4)	36 (61.0)	1.00	1.00	1.00	1.00
Yes	150 (35.5)	83 (43.0)	23 (39.0)	1.35 (0.95–1.92)	1.21 (0.81–1.80)	1.11 (0.64–1.95)	1.17 (0.63–2.18)
Missing	12 (2.8)	3 (1.6)	0 (0.0)				
*Beer *							
Preconception							
No	273 (64.5)	108 (56.0)	38 (64.4)	1.00	1.00	1.00	1.00
Yes	135 (32.0)	82 (42.5)	21 (35.6)	1.54 (1.08–2.19)	1.36 (0.91–2.03)	1.12 (0.63–1.98)	1.13 (0.60–2.14)
Missing	15 (3.5)	3 (1.5)	0 (0.0)				
≤1 glass/week	46 (10.9)	32 (16.6)	12 (20.3)	1.84 (1.12–3.04)	1.30 (0.71–2.36)	1.87 (0.91–3.84)	2.06 (0.90–4.76)
>1 glass/week	66 (15.6)	39 (20.2)	6 (10.2)	1.44 (0.91–2.28)	1.47 (0.88–2.46)	0.65 (0.26–1.60)	0.75 (0.29–1.94)
Missing	38 (9.0)	14 (7.2)	3 (5.1)				
				*P* trend = 0.03		*P* trend = 0.68	
During pregnancy							
No	337 (79.7)	146 (75.6)	52 (88.1)	1.00	1.00	1.00	1.00
Yes	73 (17.3)	44 (22.8)	7 (11.9)	1.39 (0.91–2.12)	1.29 (0.78–2.14)	0.62 (0.27–1.43)	0.84 (0.35–2.00)
Missing	13 (3.0)	3 (1.5)	0 (0.0)				
≤1 glass/week	27 (6.4)	21 (16.6)	4 (6.8)	1.80 (0.98–3.28)	1.13 (0.50–2.52)	0.96 (0.32–2.86)	1.38 (0.43–4.42)
>1 glass/week	34 (8.0)	19 (20.2)	1 (1.7)	1.29 (0.71–2.34)	1.43 (0.74–2.77)	0.19 (0.03–1.42)	0.21 (0.28–1.63)
Missing	25 (5.9)	7 (3.6)	2 (3.4)				
				*P* trend = 0.17		*P* trend = 0.07	
*Spirits *							
Preconception							
No	397 (93.9)	178 (92.2)	51 (86.4)	1.00	1.00	1.00	1.00
Yes	12 (2.8)	4 (2.1)	3 (5.1)	0.74 (0.24–2.34)	0.84 (0.22–3.29)	1.95 (0.53–7.13)	3.61 (0.83–15.7)
Missing	14 (3.3)	11 (5.7)	5 (8.5)				
≤1 glass/week	4 (0.9)	2 (1.1)	1 (1.7)	1.11 (0.20–6.14)	0.79 (0.08–7.90)	1.95 (0.21–17.8)	2.06 (0.17–24.4)
>1 glass/week	5 (1.2)	0 (0.0)	2 (3.4)	0 (0.0)	0 (0.0)	3.11 (0.59–16.5)	7.18 (1.09–47.3)
Missing	17 (4.0)	13 (6.7)	5 (8.5)				
				*P* trend = 0.14		*P* trend = 0.21	
During pregnancy							
No	397 (93.9)	182 (94.3)	53 (89.8)	1.00	1.00	1.00	1.00
Yes	12 (2.8)	0 (0.0)	1 (1.7)	0 (0.0)	0 (0.0)	0.62 (0.08–4.90)	0.87 (0.10–7.55)
Missing	14 (3.3)	11 (5.7)	4 (6.8)				
≤1 glass/week	5 (1.2)	0 (0.0)	0 (0.0)	0 (0.0)	0 (0.0)	0 (0.0)	0 (0.0)
>1 glass/week	2 (0.4)	0 (0.0)	1 (1.7)	0 (0.0)	0 (0.0)	3.75 (0.33–42.0)	9.13 (0.61–135)
Missing	19 (4.5)	0 (0.0)	5 (8.5)				
						*P* trend = 0.88	
*Other beverage* ^b^							
Preconception							
No	376 (88.9)	164 (85.0)	49 (83.0)	1.00	1.00	1.00	1.00
Yes	33 (7.8)	18 (9.3)	5 (8.5)	1.25 (0.68–2.29)	1.48 (0.74–2.96)	1.16 (0.43–3.12)	2.16 (0.74–6.35)
Missing	14 (3.3)	11 (5.7)	5 (8.5)				
≤1 glass/week	18 (4.2)	13 (6.7)	4 (6.8)	1.65 (0.79–3.46)	1.93 (0.84–4.44)	1.70 (0.55–5.24)	3.42 (0.81–14.3)
>1 glass/week	13 (3.1)	4 (2.1)	1 (1.7)	0.71 (0.23–2.20)	0.93 (0.28–3.16)	0.59 (0.07–4.61)	1.26 (0.14–11.5)
Missing	14 (3.3)	12 (6.2)	5 (8.5)			
				*P* trend = 0.91		*P* trend = 0.89	
During pregnancy							
No	385 (91.0)	174 (90.2)	54 (91.5)	1.00	1.00	1.00	1.00
Yes	24 (5.7)	8 (4.1)	4 (6.8)	0.74 (0.36–1.67)	0.94 (0.37–2.40)	1.28 (0.43–3.85)	2.26 (0.68–7.51)
Missing	14 (3.3)	11 (5.7)	1 (1.7)				
≤1 glass/week	13 (3.1)	4 (2.1)	3 (5.1)	0.68 (0.22–2.12)	0.99 (0.28–3.46)	1.78 (0.49–6.45)	3.41 (0.81–14.3)
>1 glass/week	7 (1.7)	3 (1.5)	1 (1.7)	0.95 (0.24–3.71)	1.19 (0.27–5.19)	1.10 (0.13–9.13)	1.26 (0.14–11.6)
Missing	18 (4.2)	12 (6.2)	1 (1.7)				
				*P* trend = 0.57		*P* trend = 0.82	

^a^Adjusted OR by use of oral contraceptives during pregnancy, maternal age at child birth, maternal education, birth weight, and infant ethnicity, three months before pregnancy, including wine consumption; ALL, acute lymphoblastic leukemia; AML, acute myeloid leukemia; *n* = number of cases; Adj. OR, adjusted odds ratio; CI, confidence interval; one glass = 200 mL.

^b^Preconception (legend: three months before pregnancy) and ^c^other beverages (legend: including wine consumption).

**Table 3:** Maternal alcohol drinking preconception and/or during pregnancy risk according to offspring age strata, leukemia subtypes, and control mothers, Brazil, 1999–2007.

Maternal drinking	Controls n (%)	ALL n (%)	AML n (%)	ALL		AML	
				OR (95% CI)	Adj.^a^ OR (95% CI)	OR (95% CI)	Adj.^a^ OR (95% CI)
*Any beverages* ≤11 mo. No	158 (37.3)	47 (24.3)	18 (30.5)	1.00	1.00	1.00	1.00
Yes	89 (21.1)	40 (20.7)	10 (17.0)	1.52 (0.92–2.48)	1.29 (0.73–2.27)	0.99 (0.44–2.23)	0.97 (0.38–2.46)
Missing	8 (1.9)	1 (0.5)	0 (0.0)				
12–23 mo. No	103 (24.4)	60 (31.1)	18 (30.5)	1.00	1.00	1.00	1.00
Yes	61 (14.4)	43 (22.3)	13 (22.0)	1.21 (0.73–2.00)	1.07 (0.61–1.89)	1.22 (0.56–2.66)	1.49 (0.61–3.61)
Missing	4 (0.9)	2 (1.1)	0 (0.0)				
*Preconception* ≤11 mo.							
No	158 (37.3)	45 (23.3)	18 (30.5)	1.00	1.00	1.00	1.00
Yes	86 (20.3)	42 (21.7)	10 (17.0)	1.72 (1.04–2.82)	1.56 (0.88–2.79)	1.02 (0.45–2.31)	1.01 (0.39–2.61)
Missing	11 (2.7)	1 (0.5)	0 (0.0)				
12–23 mo.							
No	103 (24.4)	60 (31.1)	18 (30.5)	1.00	1.00	1.00	1.00
Yes	61 (14.4)	43 (22.3)	13 (22.0)	1.21 (0.73–2.00)	1.01 (0.57–1.77)	1.22 (0.56–2.66)	1.39 (0.59–3.27)
Missing	4 (0.9)	1 (0.5)	0 (0.0)				
*During pregnancy* ≤11 mo.							
No	205 (48.4)	62 (32.1)	24 (40.7)	1.00	1.00	1.00	1.00
Yes	50 (11.8)	26 (13.5)	4 (6.8)	1.72 (0.99–2.99)	1.49 (0.77–2.89)	0.68 (0.23–2.06)	0.96 (0.30–3.10)
Missing	0 (0.0)	1 (0.5)	0 (0.0)				
12–23 mo.							
No	129 (30.5)	83 (43.0)	25 (42.4)	1.00	1.00	1.00	1.00
Yes	37 (8.8)	21 (10.9)	6 (10.1)	0.89 (0.49–1.63)	0.83 (0.40–1.68)	0.84 (0.32–2.19)	1.09 (0.38–3.11)
Missing	2 (0.5)	0 (0.0)	0 (0.0)				

^a^Adjusted odds ratio by use of oral contraceptives during pregnancy, maternal age at birth, maternal education, birth weight, and infant skin color; ^b^three months before pregnancy; ALL, acute lymphoblastic leukemia; AML, acute myeloid leukemia; *n* = number of cases; Adj. OR, adjusted odds ratio; CI, confidence interval; mo. = months.

^b^Preconception (legend: three months before pregnancy).

**Table 4:** Maternal smoking and alcohol consumption interaction risk for early age leukemia, Brazil, 1999–2007.

Type of analysis/smoke burden^b^	Alcohol consumption^a^					
	ALL			AML		
	Abstainers	Occasional drinkers	Frequent drinkers	Abstainers	Occasional drinkers	Frequent drinkers
*Periconception *						
Crude OR (95% CI)						
Nonsmokers	1.00	1.67 (0.96–2.94)	1.14 (0.63–2.06)	1.00	2.01 (0.92–4.36)	0.97 (0.38–2.47)
Moderate smokers	0.64 (0.36–1.12)	0.00	2.10 (0.77–5.78)	0.66 (0.28–1.58)	1.56 (0.18–7.60)	0.45 (0.04–4.67)
Heavy smokers	2.10 (0.69–6.44)	1.78 (0.51–6.17)	1.36 (0.20–9.53)	0.99 (0.19–8.34)	0.00	0.00
Adj.^c^ OR (95% CI)						
Nonsmokers	1.00	0.88 (0.39–1.98)	1.22 (0.59–2.53)	1.00	2.01 (0.81–4.96)	1.19 (0.43–3.27)
Moderate smokers	1.00 (0.51–1.94)	0.00	1.46 (0.48–4.43)	0.64 (0.25–1.66)	1.28 (0.16–10.2)	0.38 (0.03–4.29)
Heavy smokers	1.89 (0.28–12.69)	0.97 (0.22–4.29)	1.41 (0.16–12.3)	1.16 (0.13–10.5)	0.00	0.00
*During pregnancy *						
Crude OR (95% CI)						
Nonsmokers	1.00	1.24 (0.63–2.45)	1.19 (0.62–2.30)	1.00	1.63 (0.67–3.98)	0.21 (0.03–1.58)
Moderate smokers	1.00 (0.55–1.81)	1.69 (0.44–6.45)	0.91 (0.23–3.55)	0.44 (0.13–1.48)	0.00	0.00
Heavy smokers	2.39 (0.47–12.0)	0.00	1.68 (0.09–32.3)	2.10 (0.21–20.6)	0.00	0.00
Adj.^c^ OR (95% CI)						
Nonsmokers	1.00	0.88 (0.39–1.98)	1.22 (0.59–2.53)	1.00	1.78 (0.66–4.80)	0.22 (0.03–1.71)
Moderate smokers	1.00 (0.51–1.94)	1.26 (0.26–6.16)	1.00 (0.23–4.44)	0.35 (0.10–1.31)	0.00	0.00
Heavy smokers	1.89 (0.28–12.69)	0.00	1.61 (0.05–51.0)	3.47 (0.32–37.0)	0.00	0.00

^a^OR, odds ratios, and 95% CI (confidence intervals) for leukemia according to categories of joint tobacco and alcohol exposures comparing models that assuming independence of effects and effect modification; level of pregnancy alcohol consumption: abstainers (0 glasses/week); occasional drinkers (≤1 glass/week); frequent drinkers (≥1 glass/week); ^b^level of pregnancy smoke consumption: nonsmokers; moderate smokers (1–≤20 cigarettes/day); heavy smokers (≥20 cigarettes/day); ALL, acute lymphoblastic leukemia; AML, acute myeloid leukemia; *n* = number of cases; Adj. OR, adjusted odds ratio; CI, confidence interval; ^c^adjusted OR by use of oral contraceptives during pregnancy, maternal age at birth, maternal education, birth weight, and infant skin color.
